# Genometrics as an essential tool for the assembly of whole genome sequences: the example of the chromosome of *Bifidobacterium longum *NCC2705

**DOI:** 10.1186/1471-2180-5-60

**Published:** 2005-10-13

**Authors:** Lionel Guy, Dimitri Karamata, Philippe Moreillon, Claude-Alain H Roten

**Affiliations:** 1 Département de Microbiologie Fondamentale, Faculté de Biologie et Médecine, Université de Lausanne, CH-1015 Lausanne, Switzerland

## Abstract

**Background:**

Analysis of the first reported complete genome sequence of *Bifidobacterium longum *NCC2705, an actinobacterium colonizing the gastrointestinal tract, uncovered its proteomic relatedness to *Streptomyces coelicolor *and *Mycobacterium tuberculosis*. However, a rapid scrutiny by genometric methods revealed a genome organization totally different from all so far sequenced high-GC Gram-positive chromosomes.

**Results:**

Generally, the cumulative GC- and ORF orientation skew curves of prokaryotic genomes consist of two linear segments of opposite slope: the minimum and the maximum of the curves correspond to the origin and the terminus of chromosome replication, respectively. However, analyses of the *B. longum *NCC2705 chromosome yielded six, instead of two, linear segments, while its *dnaA *locus, usually associated with the origin of replication, was not located at the minimum of the curves. Furthermore, the coorientation of gene transcription with replication was very low.

Comparison with closely related actinobacteria strongly suggested that the chromosome of *B. longum *was misassembled, and the identification of two pairs of relatively long homologous DNA sequences offers the possibility for an alternative genome assembly proposed here below. By genometric criteria, this configuration displays all of the characters common to bacteria, in particular to related high-GC Gram-positives. In addition, it is compatible with the partially sequenced genome of DJO10A *B. longum *strain. Recently, a corrected sequence of *B. longum *NCC2705, with a configuration similar to the one proposed here below, has been deposited in GenBank, confirming our predictions.

**Conclusion:**

Genometric analyses, in conjunction with standard bioinformatic tools and knowledge of bacterial chromosome architecture, represent fast and straightforward methods for the evaluation of chromosome assembly.

## Background

*Bifidobacterium longum *is an obligate anaerobe, belonging to the *Actinomycetales*, a branch of the high-GC Gram-positive bacteria which includes, among others, corynebacteria, mycobacteria and streptomycetes. *B. longum *is a natural colonizer of the gastrointestinal tract (GIT) and the vagina [1]. It is one of the very first bacteria which colonize the sterile GIT of newborns, predominating in breast-fed infants until weaning [2]. Thereafter, its numerical importance decreases, while *Bacteroides *and other taxa replace it [3]. *B. longum*, a harmless bacterium, considered to play an important role in maintaining a healthy GIT by preventing diarrhea, improving lactose intolerance, and participating to immunomodulation [1], is now widely used in health-promoting foods.

Recently, the whole genome of *B. longum *strain NCC2705 has been sequenced [4]. Comparison with other high-GC Gram-positives revealed high levels of protein homology with *Streptomyces coelicolor *A3(2) (34% of best hits), *Mycobacterium tuberculosis *(9.3% of best hits) and, to a lesser extent, with other actinobacteria as well as with some unrelated genera such as *Clostridium *and *Streptococcus*. Surprisingly, it contains a very high number of genetic entities related to mobile elements such as transposons and plasmids. There are 14 integrases/recombinases, 16 intact insertion sequences (ISs), many remnants of ISs, one integrated plasmid, many possible remnants of integrated plasmids and prophages. The origin and the terminus of chromosome replication of *B. longum *NCC2705 could not be accurately localized along the initial whole genome sequence. Today, DJO10A, another strain of *B. longum*, is almost fully sequenced, but not assembled.

The presence of many ISs and IS remnants in *B. longum *NCC2705 leaves open the possibility of major chromosomal rearrangements [4]. These internal recombination events were already advanced to explain the poor conservation of gene order during the evolution of prokaryotic genomes [5].

It appears that major chromosomal rearrangements almost always consist in inversions of a segment of the chromosome centered on the origin of replication [6,7]. Other inversions are probably counter-selected [8], since, unlike inversions around the origin of replication, they change the orientation of transcription relative to DNA replication or they change the length of chromosome arms. Such events have adverse effects on both replication speed and transcription [9,10]. Alternatively, it has been proposed that rearrangements preferentially centered on the origin of replication are favored by the bidirectional DNA replication: starting simultaneously from the origin of chromosome replication, the two replication forks are at the same distance from it and are likely to be in close contact [6]. Thus, DNA breaks produced by topoisomerases would generate structures suitable for recombinations between the two chromosomal arms, leading to origin-centered rearrangements [6].

The coorientation between gene transcription and DNA replication is apparently a fundamental feature of bacterial chromosome architecture. More specifically, ORFs and tRNA genes follow a similar tendency and all so far identified ribosomal RNA operons are cooriented with DNA replication [11,12]. The asymmetric bias in the nucleotide composition at the genome level is another relevant feature (for a review see [13]). The leading strand, defined by chromosome replication, is generally enriched in guanines (Gs) and depleted in cytosines (Cs). To explain this observation several proposals have been advanced: (i) a preferential usage of certain codons to avoid frameshifting during translation [14], (ii) the enrichment of coding sequences in purines so as to avoid mRNAs secondary structures [15,16], (iii) mutational biases targeting single-stranded DNA present during transcription [17] or (iv) during DNA replication [18]. Mechanisms that would lead to the observed biases in models (i) to (iii) rest on the widespread coorientation of gene transcription and chromosome replication (see above). These asymmetric biases have allowed to unambiguously determine the origin of replication in almost all bacteria [12,18-20] as well as the terminus of replication in a large majority of the species [12].

Genomic rearrangements are often highlighted by comparison of whole chromosomal sequences belonging to the same species or genus. For example, dot-plot analyses revealed two recombination events in *Streptococcus pyogenes *SSI-1, with respect to other *S. pyogenes *strains, leading to an inversion around the origin of replication [21]. Since they do not change the orientation of transcription relative to DNA replication, these symmetric rearrangement were not revealed by nucleotide bias (skew) analysis.

However, several examples of asymmetric rearrangements are known, pointed out by nucleotide skew analyses. Several isolates of the original clone of *Pseudomonas aeruginosa *PAO1, a high-GC gamma-proteobacterium and an opportunistic pathogen, have an inversion of a third of their chromosome. The inversion occurs by homologous recombination between two rRNA loci: *rrnA *and *rrnB*. As a consequence, the circular chromosome is divided into two inequal arms of one and two thirds, instead of the usual two halves [22]. This asymmetry is obvious on a cumulative GC- or TA skew curve (see the *P. aeruginosa *PAO1 page on Comparative Genometrics website [23]). The citrus pathogen *Xylella fastidiosa *9a5c is another example of asymmetric rearrangements, that is highlighted when compared with another *X. fastidiosa *strain, Temecula1 [24,25]. In this case, the rearrangements occur between three pairs of prophage-related elements [25], also dividing the chromosome of strain 9a5c in two arms of inequal lenghts (one third and two thirds), as in *P. aeruginosa *PAO1 (see pages for *X. fastidiosa *strains on Comparative Genometrics [23]). In *Yersinia pestis *strains, the high number of insertion sequence (IS) copies leads to frequent recombination events, inverting segments of the chromosome and changing their orientation of transcription with respect to replication. These inversions are easily spotted on a GC skew plot (see pages for *Y. pestis *strains on Comparative Genometrics [23]). In all three above cases, the rearrangements occur naturally, and do not constitute an incorrect genome assembly.

In this contribution we assess the assembly of the initially deposited genome sequence of *B. longum *NCC2705 by genometric methods, rapid and efficient tools suitable for testing the assembly of prokaryotic chromosomes [23]. Our analysis, strongly suggesting that the chromosome of *B. longum *NCC2705 was initially misassembled, was confirmed by Schell *et al*. [26] during the review of this contribution.

## Results and discussion

### Analysis of the initial *Bifidobacterium longum *NCC2705 genome sequence

Investigation with genometric tools of the initially released nucleotide sequence of *B. longum *NCC2705 (Configuration I [GenBank:NC_004307.1]) revealed several atypical features.

**Figure 1:**
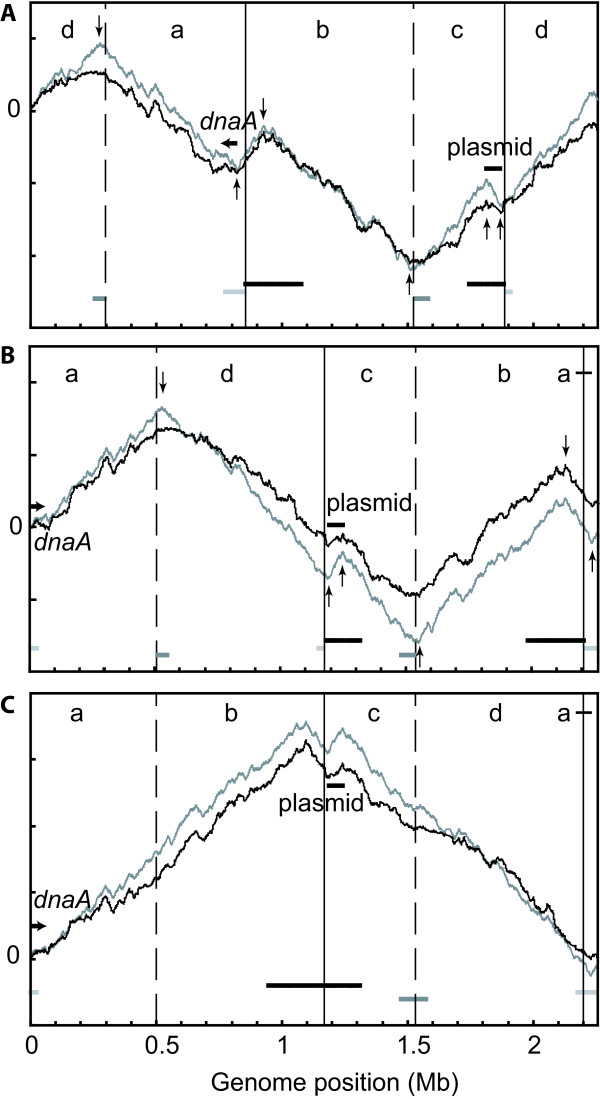
**Genometric analyses of the two chromosomal configurations of *B. longum *NCC2705. **Cumulative GC- and ORF orientation skew analyses of chromosomal configurations I (A, B) and II (C) of *B. longum *NCC2705. Configuration I [GenBank:NC_004307.1] is plotted without modification with respect to the version available on databases (Watson strand), (A) as well as the Crick strand of same sequence starting with the gene *dnaA *(B). Cumulative GC skew of the first codon position (black curve) and cumulative ORF orientation skew (grey curve) are plotted as a function of their position on the genome. Distance between two graduation marks represents an excess of 10^4 ^Gs over Cs in the first codon position cumulative GC skew and of 2·10^3 ^nucleotides in the ORF orientation skew. Thin arrows indicate relevant changes of the slope sign. The thick arrow corresponds to the position and the orientation of *dnaA*. The location of the integrated plasmid described in [4] is indicated. Vertical lines specify the position of copies of ISBlo2 (dashed line) and ISBlo5 (full line) insertion sequences. Segments delimited by these insertion sequences are designated a, b, c and d. Horizontal lines below the curves are the hits of scaffolds 1 (black), 8 (light grey) and 9 (dark grey) of *B. longum *DJO10A when BLASTed against the *B. longum *NCC2705 genome in configurations I (A, B) or II (C).

First, cumulative GC skew on the first codon position, as well as the cumulative ORF orientation skew, yielded a curve with six significant changes of the slope sign. Furthermore, the *dnaA *gene was not located at the lowest minimum of the curve (Figure 1A and 1B). This is clearly different from all known high-GC Gram-positives (see [23,27]), since they essentially exhibit one maximum and one minimum, the latter being generally located in the vicinity of the *dnaA *gene [12].

The presence of *dnaA *at a place other than the minimum of the cumulative ORF orientation curve has never been reported in high-GC Gram-positive bacteria. For a large majority of bacterial species, it was shown that *dnaA*, a gene whose product binds to the origin of replication and participates in the initiation of replication, is located close to the origin [12,28]. More generally, in archaea, gene *orc1/cdc6*, which encodes the archaeal counterpart of DnaA, is very often also located close to the origin of chromosome replication. Finally, in sequenced genomes, half of archaea and most of bacterial genes encoding origin binding proteins are close to the minimum of the cumulative GC skew and ORF orientation curves [12].

Second, in the first published *B. longum *NCC2705 sequence, coorientation indexes (CI), i.e. the proportion of genes or of a given subset of genes that are transcribed in the direction of chromosome replication, revealed significant anomalies. Indeed, the CI of protein encoding genes and tRNAs was 0.48 and 0.47, respectively, while only one out of the four rRNA operons was cooriented (Table 1). These low CIs are most uncommon in bacteria where it has been shown that the majority of protein encoding genes [11] as well as of tRNA genes are cooriented with chromosome replication (i.e. CIs higher than 0.5), while, so far, a strict coorientation of rRNA operons constitutes a universal rule in prokaryotes [12]. More specifically, CIs of protein encoding genes of *M. tuberculosis *CDC1551 and *S. coelicolor *A3(2), two high G+C Gram-positives related to *B. longum *NCC2705, are 0.59 and 0.55, respectively. CIs of tRNAs of the same species are similar, i.e. 0.62 and 0.57, respectively, whereas all rRNA operons are cooriented (Table 1).

**Figure 2:**
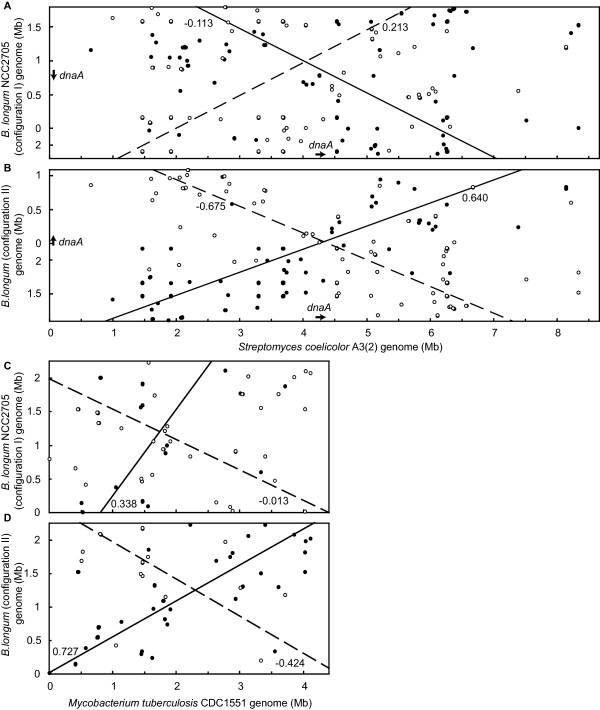
**Between-species comparisons of the two chromosomal configurations of *Bifidobacterium longum *NCC2705 and of related species. **Relative positions of homologous segments on sequences of configurations I (A, C) and II (B, D) of *B. longum *NCC2705 against *S. coelicolor *A3(2) (A, B) and *M. tuberculosis *CDC1551 (C, D). Full circles and open circles indicate pairs of homologous DNA segments that have the same, respectively the opposite orientation on the chromosome. Since the *S. coelicolor *A3(2) genome is linear, its origin of replication cannot be placed at position 1. Therefore, in A and B, both configurations of the *B. longum *genome are linearized by placing the terminus of replication at the origin of the graph. For *B. longum *NCC2705 in configuration I (A), the probable terminus of replication of the integrated plasmid has been chosen as the terminus of replication and placed at the origin of the graph. For the *B. longum *in configuration II, the maximum of the first codon position cumulative GC skew has been chosen as the terminus. On (A) and (B), position and orientation of the *dnaA *gene on each genome is indicated by a thick arrow. Lines represent type II regressions of direct (full line) and of indirect hits (dashed line). For each regression, the corresponding correlation coefficient is indicated.

Third, between-species whole-genome alignments of *B. longum *NCC2705 and *M. tuberculosis *CDC1551 or *S. coelicolor *A3(2) revealed a very poor conservation of gene order (Figure 2) [6,7]. Indeed, correlation coefficients measuring the conservation of gene order between species are close to zero, whereas in the ideal case the coefficient of correlation should be 1 and -1 for the direct and respectively indirect homologous DNA segment subsets.

In summary, genometric analyses revealed major anomalies in the organization of the *B. longum *NCC2705 genome: (i) several changes in the sign of the slope of the cumulative nucleotide skew curves, and location of the *dnaA *gene far from the minimum of the curve, (ii) low gene coorientation indexes and (iii) absence of correlation between *B. longum *NCC2705 and related species in between-species whole-genome alignments.

### Relationship of *B. longum *strains NCC2705 and DJO10A

Availability of numerous contigs of the genome of DJO10A, another *B. longum *strain, strongly suggested that the initially reported sequence of NCC2705 chromosome could have been incorrectly assembled, or had undergone major chromosomal rearrangements. Indeed, BLAST results reveal three DJO10A long scaffolds -number 1, 8 and 9 – each with a large number of hits in two different regions of the *B. longum *NCC2705 chromosome (Figure 1A and 1B). These homology discontinuities occur in four regions encompassing extrema of the cumulative skew curves (Figure 1A). Within each of these four regions, an insertion sequence was identified: ISBlo2a and ISBlo2b, belonging to the IS21 family, and ISBlo5c and ISBlo5d, belonging to the IS256 family (Figure 1A, 1B and Table 2)[4].

**Figure 3:**
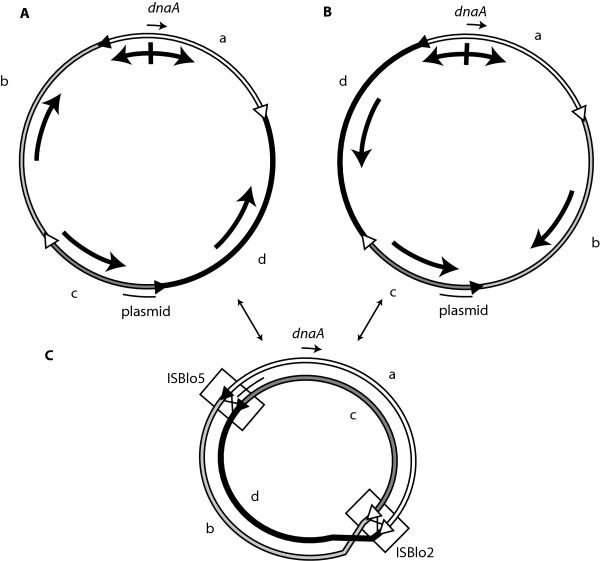
**Chromosome rearrangements generating the transition between the two *Bifidobacterium longum *configurations. **Maps of *B. longum *NCC2705 in configurations I (A), II (B) and the proposed mechanism mediating the transition between the two configurations (C). Segments a, b, c and d are indicated. Gene *dnaA *and the integrated plasmid are represented by an arrow and a thin black line, respectively. ISBlo2a and b are indicated by open triangles, ISBLo5c and d by solid ones. For each segment, thick black arrows indicate the orientation of transcription of the majority of the genes.

The presence of these insertion sequences (Table 2) at putative recombination sites offers a straightforward way to account for chromosomal rearrangements which can mediate the shift between initial configuration of strain NCC2705 and the putative configuration of strain DJO10A, here below designated configurations I and II, respectively (Figure 3). Indeed, translocation and inversion of the two large segments a and d, achieved by two homologous recombination events between ISBlo2a and 2b, on one side, and ISBlo5c and 5d, on the other side, would allow the interconversion between configurations I and II. This assumption is supported by the cross-exchange of direct repeats in both IS pairs, as noted in the IS Finder database [29].

### Analysis of the configuration II of the *B. longum *NCC2705 chromosome

Genometric analyses of the *B. longum *NCC2705 chromosome in configuration II reveal a genome architecture typical of high-GC Gram-positive organisms. Indeed, the cumulative GC-skew curve performed on the first codon positions and the cumulative ORF orientation skew curves are very similar to those characteristic of high-GC Gram-positive chromosomes (Figure 1C). First, both skew curves exhibit essentially one minimum and one maximum corresponding, respectively, to the origin and the terminus of chromosome replication; *dnaA *being at the minimum of the curve while the integrated plasmid is close to the probable terminus of replication, at the maximum of the skew curves. The two minor peaks correspond to the extremities of the integrated plasmid. This genetic element is antioriented, so that the majority of its genes are transcribed in the opposite direction with respect to chromosome replication, leading to a short reversal both in the cumulative ORF orientation skew and the cumulative first codon GC skew curves. This fact was already reported for other integrated elements (for example in *Parachlamydiaceae *UWE25 [30]) and is possibly a consequence of the instability of the integration. Integration of foreign DNA stretches close to the terminus is common, for example in prophages [25]. A higher frequency of recombination at the terminus of replication was proposed as the source of this instability [31,32]. It appears that of the six changes in the sign of the skew slopes of strain NCC2705 in configuration I, one does actually correspond to the origin of chromosome replication and a recombination site, three to other recombination sites, one to the likely terminus of replication, and the last one to the distal extremity of an integrated plasmid. Second, in configuration II, the CIs of protein encoding- and tRNA genes are 0.66 and 0.61, respectively, while all rRNA operons are cooriented, a situation characteristic of prokaryotes (Table 1). Third, between-species whole-genome alignments of *B. longum *in configuration II and *S. coelicolor *A3(2) or *M. tuberculosis *CDC1551 display a fair conservation of gene order (Figure 2C, 2D). Correlation coefficients of type II regressions for direct and indirect homologous DNA segment subsets are much closer to 1 or -1, respectively, than those obtained with the sequence of *B. longum *NCC2705 in configuration I (Figure 2C, 2D). Finally, each of the scaffolds 1, 8 and 9 of *B. longum *DJO10A has hits in one single region of the *B. longum *NCC2705 chromosome in configuration II (Figure 1C).

A genome sequence corresponding approximatively to configuration II of *B. longum *NCC2705 [GenBank:NC_004307.2] has been recently deposited in the GenBank database by Schell et al. [26]. Whereas we hypothesized that the two pairs of IS, ISBlo2a, ISBlo2b, ISBlo5c and ISBlo5d were the only four chromosomal rearrangement loci, these authors found experimental evidences that, moreover, the initial sequence of NCC2705 had been misassembled at the level of the three ribosomal RNA operons. The sequence in configuration II proposed in this contribution has three DNA segments (totalizing 226 kb, i.e. 10% of the genome) which are differently assembled than the corrected sequence. These assembly discrepancies have only very limited consequences on the results of our analyses.

Thus, configuration II, similar to the recently deposited sequence of the *B. longum *NCC2705 genome [Genbank:NC_004307.2], is endowed with all chromosomal features common to high-GC Gram-positive bacteria: (i) cumulative GC-and ORF orientation skew curves are typical and the *dnaA *gene is located at the minimum of the curves, (ii) between-species whole-genome alignments provide the expected X-shape and the coefficients of correlation are relatively close to 1 or -1 and (iii) relevant contigs of *B. longum *DJO10A are each homologous to a single continuous region of the proposed NCC2705 chromosome.

## Conclusion

Genometric analyses – nucleotide skews, coorientation indexes, BLAST comparisons and between-species whole-genome alignments – revealed a most peculiar chromosomal architecture of the initially reported sequence of the *B. longum *NCC2705 genome. This observation may have two explanations.

First, it is highly probable that in the final stages of the sequencing process, the genome of *B. longum *NCC2705 was misassembled, a possibility presently favored by Schell and coworkers, in particular since independently performed long-range PCR experiments confirm the presence of configuration II, but could not detect configuration I (F. Arigoni, personal communication). This is in full agreement with genometric analyses of more than 150 genome sequences ([23] and supplementary material of Tillier and Collins [20,33]) which reveal a near universal architecture of prokaryotic chromosomes, also found in configuration II of *B. longum *NCC2705.

Second, the genome of NCC2705 may possibly undergo major chromosomal rearrangements, yielding either of the two alternative configurations I and II. The interconversion between them, achieved by two crossovers between two pairs of homologous insertion sequences (IS), would have drastic consequences. In particular, in configuration II, the cooriented transcription of the majority of the genes, including all rRNA operons, with chromosome replication would allow higher growth rates in suitable conditions. Absence of significant coorientation in configuration I due to the inversion of large segments b and d, representing about 50% of the chromosome, would probably considerably increase the generation time because of collisions between the DNA- and the RNA polymerase [9]. However, as discussed here above, the adverse effects of inversions, other than those around the origin of replication, would render the existence of configuration I highly unlikely. Actually, the latter has apparently not been detected in long range PCR experiments (F. Arigoni, personal communication). However, inversions of relatively long segments – leading to the antiorientation of the majority of the genes in the segment – have been reported, for example, in *Yersinia pestis *[34-37], and thus may not be completely excluded in *B. longum *NCC2705.

For the first time, our analyses illustrate the potential of fast and straightforward genometric methods to test genome assembly. They almost immediately revealed gross anomalies of the *B. longum *NCC2705 initially published sequence, pointing to an incorrect assembling. In conclusion, although their results have to be supported by experimental verification, these simple and powerful tools are essential for the assembly of a chromosome sequence, and for its final validation.

## Methods

### Sequences

Full genome sequences and annotation files of *B. longum *NCC2705,*S. coelicolor *A3(2), *M. tuberculosis *CDC1551 and contigs of an unfinished sequence of *B. longum *DJO10A were retrieved from NCBI database [38,39]. For *B. longum *NCC2705, the initial [GenBank:NC_004307.1] and the second [GenBank:NC_004307.2] versions of the chromosome (released on August, 27^th^, 2002 and on January, 21^st^, 2005 respectively) were downloaded. Sequence of configuration II as proposed in this contribution, and both initial and corrected versions of the *B. longum *NCC2705 genome are available in fasta format [40]. As proposed by Cebrat et al. [41], we term the genome sequences available on databases and those of the complementary strands the Watson- and Crick strands, respectively.

### Genome analyses

Genomes were investigated by cumulative genomic GC skew, first codon position GC skew, and ORF orientation skew [19,20,42]. We used the algorithms described in [27,30] and implemented in the Genometrician's Scooter [43].

### Nucleotide skews

As defined by Lobry [18], a GC skew is the difference between the number of Gs and Cs normalized to the G+C content. In our contribution we used the non-normalized nucleotide skew, calculated in 1-kb windows along the genome. In the genomic GC skew, the whole genome sequence is used. For GC skew on the first codon position, only nucleotides at the first position of codons are considered for the skew calculation.

### Cumulative nucleotide skews

Slightly different from the definition of Grigoriev [19], the cumulative nucleotide skew of any given window is the nucleotide skew of the latter (see above) added to the sum of skews of all preceding windows.

### Cumulative ORF orientation skews

As in [20], in the ORF orientation skew analysis, the value attributed to each ORF corresponds to its length, considered as positive if the ORF is located on the Watson strand, and negative if encoded on the Crick strand. The cumulative ORF orientation analysis is calculated as a cumulative nucleotide skew by replacing windows and GC skews by genes and ORF orientation skews: the value corresponding to a given ORF is added to the sum of the values of all upstream-located ORFs. A cumulative ORF orientation skew is represented as a function of the position of the center of each gene. We used the number of nucleotide per gene, and not the number of ORFs to normalize the signal to the length of the gene, otherwise, in the cumulative ORF orientation skew plot, small genes would have a greater importance than long ones.

### Coorientation Indexes (CI)

For all genomes, coorientation indexes (CI), i.e. the percentage of all or of certain categories of genes – protein encoding genes, rRNAs, tRNAs – transcribed in the direction of DNA replication, were calculated according to [12]. For that purpose, the origin and the terminus of chromosome replication are determined by cumulative GC skew. For *B. longum *NCC2705, where the cumulative GC skew did not reveal the origin and/or the terminus of replication, the first codon position cumulative GC skew and ORF orientation skews were used. In most so far sequenced bacterial genomes, the origin of replication is located at the minimum of the cumulative skew curves. *S. coelicolor *A3(2), that has an extremely high G+C content, is an exception since its origin of replication is located at the maximum of the genomic GC skew curve. Generally, the origin of replication was shown to be close to the *dnaA *gene. The terminus of replication is assumed to be at the maximum of the skew curves, except in *S. coelicolor *A3(2), where it is assumed to be at the minimum, corresponding to both ends of the linear chromosome. However, for the first reported sequence of *B. longum *NCC2705, where the skew analyses did not provide the origin or the terminus of replication, we assumed that they are respectively located close to *dnaA *and at the putative terminus of replication in the integrated plasmid, about 180° from the *dnaA *gene on the circular chromosome.

### BLAST

Basic Local Alignment Search Tool (BLAST) 2.2.4 [44] analysis was performed with the software kindly provided by the NCBI [38] using as a cutoff an expected *E*-value of 10^-2 ^for alignments of the full genome sequence of *B. longum *NCC2705 vs. the available contigs of *B. longum *DJO10A. An *E*-value of 10^-2 ^indicates that a hit with the same or a better alignment score occurs with a probability of 10^-2 ^when searching the same database with a random sequence. BLAST results with an alignment length below 1000 nucleotides were discarded. BLAST analysis was performed with an expected *E*-value of 10 for alignments of *S. coelicolor *A3(2) and *M. tuberculosis *CDC1551 vs. *B. longum *NCC2705 in its actual as well as putative alternative chromosomal configuration. For the latter analyses only, hits with an alignment score below 100 were discarded. A hit is defined as direct or indirect if the DNA segments are in the same, or respectively opposite, orientation in both genomes.

### Between-species alignments of whole genomes

Also called dot-plot analyses [6,21], genome-to-genome comparisons were achieved according to [45]. The relative positions of homologous segments in pairwise comparisons of bacterial genomes were determined by BLAST (see above).

Correlation coefficients of type II regression (major axis regression) were determined for both direct and indirect hit subsets. If a genome had undergone only exactly symmetric rearrangements around the origin of replication, the correlation coefficients of the direct- and indirect BLAST hit sets would be 1 and -1, respectively. Correlation coefficients close to zero show no correlation between relative chromosomal positions of homologous segments.

### Accession numbers

*Bifidobacterium longum *NCC2705, [GenBank:NC_004307.1] and [GenBank:NC_004307.2]; *S. coelicolor *A3(2), [GenBank:NC_003888]; *M. tuberculosis *CDC1551, [GenBank:NC_002755]; *B. longum *DJO10A, [GenBank:NZ_AABM00000000].

## List of abbreviations

kb, kilobase; A, adenine; C, cytosine; G, guanine; T, thymine; rRNA, ribosomal RNA; tRNA, transfer RNA.

## Authors' contributions

LG carried out the analyses during his MSc thesis, supervised by CAR who designed and managed the project. LG and CAR proposed the genome configuration II. LG, DK, PM and CAR participated to the interpretation of the results. LG drafted the manuscript in collaboration with CAR and DK. All authors read and approved the final manuscript.

## Figures and Tables

**Table 1:** Coorientation indexes of different genome subsets in some high G+C Gram-positive bacteria

	ORFs	rRNA genes	tRNA genes
*B. longum *NCC2705 initial sequence	0.48	0.25	0.47
*B. longum *NCC2705 configuration II	0.66	1	0.61
*M. tuberculosis *CDC1551	0.59	1	0.62
*S. coelicolor *A3(2)	0.55	1	0.57

**Table 2:** Genetic elements on the initial sequence of *Bifidobacterium longum *NCC2705

	5' end location	3' end location	Length (nt)
ISBlo2a	295604	298058	2454
ISBlo2b	1522936	1525390	2454
*dnaA*	794998	796500	1502
ISBlo5c	853915	855280	1365
ISBlo5	1886182	1887547	1365
Integrated plasmid	1813659	1870484	56825
